# Low Impact of Fall Armyworm (*Spodoptera frugiperda* Smith) (Lepidoptera: Noctuidae) Across Smallholder Fields in Malawi and Zambia

**DOI:** 10.1093/jee/toac113

**Published:** 2022-12-14

**Authors:** Rhett Harrison, John Banda, Gilson Chipabika, Chipo Chisonga, Christopher Katema, Damian Mabote Ndalamei, Stephen Nyirenda, Howard Tembo

**Affiliations:** World Agroforestry (ICRAF), Lusaka, Zambia; Zambian Agricultural Research Institute, Mt Mukulu Research Station, Chilanga, Zambia; Zambian Agricultural Research Institute, Mt Mukulu Research Station, Chilanga, Zambia; World Agroforestry (ICRAF), Lusaka, Zambia; World Agroforestry (ICRAF), Chitedze Agricultural Research Station, Lilongwe, Malawi; Zambian Agricultural Research Institute, Mt Mukulu Research Station, Chilanga, Zambia; Department of Agricultural Research Services (DARS), Bvumbwe Agricultural Research Station, Limbe, Malawi; Zambian Agricultural Research Institute, Mt Mukulu Research Station, Chilanga, Zambia

**Keywords:** agroecology, cultural control, IPM, scouting, threshold

## Abstract

Fall armyworm (*Spodoptera frugiperda* Smith), a serious pest of cereals from the Americas, has spread across sub-Saharan Africa and Asia since 2016, threatening the food security and incomes of millions of smallholder farmers. To measure the impact of *S. frugiperda* under different management approaches, we established on-farm trials across 12 landscapes (615−1,379 mm mean annual rainfall) in Malawi and Zambia during the 2019/2020 and 2020/2021 seasons. Here we present the results from our conventional tillage, monocrop maize, no pesticide treatment, which served to monitor the background *S. frugiperda* impact in the absence of control measures. Median plot-level *S. frugiperda* incidence ranged between 0.00 and 0.52 across landscapes. Considering severe leaf damage (Davis score ≥5), the proportion of affected plants varied between 0.00 and 0.30 at the plot scale, but only 3% of plots had ≥10% severely damaged plants. While incidence and damage severity varied substantially among sites and seasons, our models indicate that they were lower in high tree cover landscapes, in the late season scouting, and in the 2020/2021 season. Yield could not be predicted from *S. frugiperda* incidence or leaf damage. Our results suggest *S. frugiperda* impacts may have been overestimated at many sites across sub-Saharan Africa. *S. frugiperda* incidence and damage declined through the cropping season, indicating that natural mortality factors were limiting populations, and none of our plots were heavily impacted. Long-term *S. frugiperda* management should be based on Integrated Pest Management (IPM) principles, including minimising the use of chemical pesticides to protect natural enemies.

Fall armyworm (*Spodoptera frugiperda* Smith) was first detected on the African continent in early 2016 (Goergen et al. 2016). It spread rapidly across sub-Saharan Africa, the Middle East and South Asia, reaching East Asia and Australia in 2018 (Nagoshi et al. 2020). *S. frugiperda* is a serious pest from the Americas, where it causes substantial damage to cereal crops especially maize and rice, and its arrival in the Old World threatens the food security and incomes of millions of smallholder farmers. In response, countries have released millions of dollars in emergency funding and donated chemical pesticides to farmers (Jepson et al. 2020). However, widespread use of toxic chemicals has serious consequences for human health and the environment (Lewis et al. 2016, Isgren and Andersson 2021, Tang et al. 2021). Poor farmers rarely use personal protective clothing, thereby exposing themselves to high levels of toxic chemicals when they spray fields, and pesticide containers are rarely disposed of properly and may even be reused as water-bottles or food containers. In addition, broad spectrum pesticides often have a much greater impact on natural enemies than on the target pest, which can lead to pest resurgence if farmers do not spray their fields multiple times a season (Hardin et al. 1995). This is particularly true for *S. frugiperda*, which feeds and hides in the maize whorl rendering many contact pesticides less effective. Furthermore, long-term use of chemical pesticides fosters a dependency, driven by their impacts on natural enemies and the development of pest resistance, leading to escalating pest management costs, which is something smallholder farmers can ill afford (Lewis et al. 1997). Hence, there is an urgent need to understand the impacts of *S. frugiperda* across the invaded range and to develop alternative pest management options that can be incorporated into a sustainable Integrated Pest Management (IPM) approach. 

*S. frugiperda* is a highly polyphagus, highly mobile, and highly fecund pest. It has been recorded feeding on >350 species in its native range (Montezano et al. 2018) and hence can persist on a wide variety of host plants when its preferred hosts, primarily maize and other poaceous plant species, are not available. The adult moths can fly continuously for over 24 h and cover over 100 km through self-powered flight (Chen et al. 2022). It is therefore capable of quickly colonising crops over a wide area soon after the seedlings emerge. Last, female *S. frugiperda* moths lay egg masses of 50–350 eggs and can lay up to 1,500 eggs over their adult life of approximately 3 wk. Under optimal temperatures (~25°C), *S. frugiperda* can complete its life-cycle within approximately 4 wk (Du Plessis et al. 2020) and hence, under suitable conditions, populations have the potential to increase rapidly. However, *S. frugiperda* is also attacked by a wide diversity of natural enemies, including parasitoids, predators, nematode parasites, entopathogenic fungi, and viruses. In its native range, around 200 species of parasitoid have been recorded attacking *S. frugiperda* (Molina-Ochoa et al. 2003) and already a substantial number of parasitoids have been recorded emerging from *S. frugiperda* in the invaded range (Sisay et al. 2018, 2019; Tendeng et al. 2019, Agboyi et al. 2020, Durocher-Granger et al. 2020). While in the Americas, *S. frugiperda* management has mostly focused on chemical pesticides and Bt corn, reports suggest that natural enemies can effectively control the pest under certain circumstances. For example, high proportions of *S. frugiperda* larvae may be attacked by parasitoids in unsprayed fields (Meagher et al. 2016) and significant mortality from fungal infections has been recorded (Guo et al. 2020). Heavy rainfall is also an important mortality factor for *S. frugiperda* (Varella et al. 2015). Young larvae are washed off leaves and older larvae drown in the whorl. Importantly, in Central America, smallholder farmers who manage biodiverse plots known as milpas, do not consider *S. frugiperda* a serious problem although it is present in most fields (Wyckhuys and O’Neil 2007). Ultimately, whether the expected yield losses surpass economic action thresholds will depend on the balance between pest pressure and natural mortality factors. Unfortunately, monitoring efforts and guidelines for action thresholds often only focus on the former (e.g., McGrath et al. 2018) and ignore the important role of natural mortality.

Since its arrival in sub-Saharan Africa, there have been a number of reports concerning the impacts of *S. frugiperda*. Most have suggested that these impacts are substantial (Kansiime et al. 2019, De Groote et al. 2020, Kassie et al. 2020, Abro et al. 2021, Tambo et al. 2021, Kwasi et al. 2022, Makale et al. 2022). For example, based on household surveys, Tambo et al. (2021) reported that severe infestations caused a 44% reduction in household income and a 17% increased likelihood of experiencing hunger in Zimbabwe. Similarly, in Kenya De Groote et al. (2020) used group discussions to assess the impact of *S. frugiperda*, and reported yield losses of 30% nationwide and over 50% in some areas. Using a similar approach in Ethiopia, Abro et al. (2021) concluded that *S. frugiperda* caused a 36% reduction in maize production nationwide. However, these estimates are all based on farmer perceptions, without any independent verification of impact estimates from field data. Even where field assessments were made many only extend to assessing infestation and leaf damage (Sisay et al. 2019), which may not be reliable predictors of yield loss (e.g., Baudron et al. 2019). Maize has a remarkable capacity to recover from leaf damage (Hruska 2019). Even under controlled screen-house conditions, it is often difficult to predict yield loss from *S. frugiperda* infestation or leaf damage estimates. In farmers’ fields the situation is made more difficult as a result of the variation in yields generated by differences in soil quality, fertilisation rates, weed management, and the effects of other pests and diseases. Although on-station trials indicate yield losses can be severe when pest pressure is high (Van den Berg et al. 2021), there are few published estimates of yield loss due to *S. frugiperda* in sub-Saharan Africa based on field measurements. Moreover, those that exist tend to suggest a lower impact than the above mentioned studies. For example, Baudron et al. (2019) surveyed 791 smallholder fields in Zimbabwe and estimated a yield loss of 11.6% from *S. frugiperda*. Quality information concerning the impact of *S. frugiperda* is critical for designing and implementing IPM strategies, as well as for allocating limited national agricultural budgets to best effect. To address this critical knowledge gap we implemented a large-scale on-farm trial across a wide climatic gradient in Malawi and Zambia. Here, we report on the results from our conventional tillage, monoculture maize, no pesticide treatment, which served to monitor the background *S. frugiperda* impact in the absence of control measures.

## Materials and Methods

### Sites

We implemented our trials across six sites (Table 1) that were chosen to represent a wide range of climatic conditions. Mean annual rainfall varied from 615 mm in Kazangula to 1,379 mm in Kawambwa (Table 1). Sites were chosen to reflect close to the full range of rain-fed conditions under which maize can be grown. At each site, we selected two landscapes, one with high tree cover and one with low tree cover. Initially the relative tree cover (i.e., high vs low) was determined through local knowledge of the sites, but this was later quantified using remote sensing (Table 1). Landscapes were approximately 30 km in diameter and in each landscape we selected 15 farms in 2019/2020 season and 12 farms in 2020/2021 season. Farms were scattered across 3–5 villages per landscape. Some trials were lost due to poor maintenance, such as inadequate weeding or in one case fire, or through the field being harvested before the researchers collected the harvest data. Hence the final number of replicates varied among landscapes (Table 1).

**Table 1.** Description of sites including mean annual rainfall, the maize variety and fertiliser inputs used, and the numbers of farmers participating in each of the high and low tree cover landscapes at each site in the 2019/2020 and 2020/2021 maize seasons

Site	Rain-fall (mm)	% Tree cover		Maize variety	Fertiliser (kg per ha)	Number of farms 2019/2020		Number of farms 2020/2021	
		Low	High			Low	High	Low	High
Kazungula	615	3.21	3.69	PAN 413	200 Comp D^*a*^ + 200 Urea	14	0	12	9
Chongwe	820	1.74	2.80	ZMS 606	200 Comp D + 200 Urea	15	12	11	12
Lilongwe	860	2.69	3.37	SC 627	150 NPK + 100 Urea	13	16	12	12
Salima	1,059	3.28	4.33	SC 627	150 NPK + 100 Urea	15	15	12	12
Thyolo	1,125	2.30	3.97	SC 537	150 NPK + 100 Urea	13	8	12	7
Kawambwa	1,379	3.99	4.01	ZMS 606	200 Comp D + 200 Urea	12	14	11	9

^*a*^N:P:K ratio 10:20:10.

### Treatment

Here, we report on our conventional tillage x monocrop maize treatment, which served as our control and monitored the background level of *S. frugiperda* impact in the absence of any management measures. There were five other treatments implemented in identical plots alongside the control. These were designed to investigate the effects of intercropping and soil amendment on *S. frugiperda* infestation, damage, and yield. No pesticides were used and we required farmers to maintain a wide pesticide-free boundary around the trial (minimum 50 m). Plots were located on smallholder farms and were surrounded by fallow agricultural land with a variable distance (but not less than 50 m) to other cultivated plots. Permanent field boundaries with shrubs and trees were a common feature, especially in the high tree cover landscapes.

Farmers were provided with inputs, including seed, basal fertiliser, and top dressing, and we assisted with the plot layout and planting to ensure consistency. The maize variety used and the fertiliser application rates varied across sites, because we wanted to choose suitable varieties and fertilisation rates for each locality (Table 1).

Maize was planted in square plots at an inter-row spacing of 0.9 m and an intra-row spacing of 0.25 m. The maize was thinned and gapped (i.e., gaps with missing plants were filled with seedlings thinned from elsewhere) approximately 10 d after emergence to maintain a standard plant density. There were 18 rows in a plot with four border rows (3.6 m); the 10 interior rows × 9 m long were used for data collection.

### Data Collection

Scouting data were collected twice each season approximately 3 wk apart. Because of the challenges of covering so many locations, the timing of scouting varied, and hence we categorised it into early and late scouting periods (3–6 wk and 6–9 wk after emergence, respectively). All data were collected from the plot core area and we used a W-format to determine data collection points. At each point on the W, we collected data from 10 plants (i.e., 50 plants total per plot), including plant height (in cm), leaf chlorophyll content (SPAD units, Konica Minolta SPAD502-plus), *S. frugiperda* infestation (Yes, No), leaf damage score using the Davis scale (Davis et al. 1992, Toepfer et al. 2021) (score 1–9), and presence of other pests and disease, such as stemborers or Maize streak virus (Yes, No). We also recorded some plot level information, such as the condition of the plot with respect to weeding. The data on plant height and leaf chlorophyll content were used to provide an objective assessment of plant health status.

At harvest, we collected data from 2 m subsamples of each row. The 2 m subsamples were off-set so that we collected a representative sample diagonally across the plot core area. For each 2 m strip, we recorded the number of plants and plant height (in cm), the number, length (in cm), and mass of cobs (in g) on each plant, the ear damage score for each cob (CIMMYT scale, score 1–9) (Prasanna et al. 2018), the raw grain weight per plot (in kg) and the adjusted grain weight per plot (in kg). To calculate the adjusted grain weight, we took a sample of grain from each plot and dried it in an oven to constant mass to calculate the moisture content and then adjusted the weight to the standard 12.5% moisture content.

### Data Analysis

Our experimental design conforms to a split-plot (2 levels of landscape tree cover per site) repeated measures (2 scouting occasions, across two seasons). Therefore, we used linear mixed-effect models to analyse our data. Our site level parameter captured variation in geographic locality, climate, and broad-scale variation in soils, as well as the maize variety used and fertilisation regimes. There was also variation among sites in the overall level of tree cover and the relative difference in tree cover between landscapes (i.e., a site:landscape interaction). For the subject level parameter we used farmer name. Thus our random term had the following form: (1 | site/landscape_type/farmer_name).

For *S. frugiperda* (%) incidence we specified a binomial model with a log-link function and we used the following independent terms: season, scouting time, landscape type, and mean annual rainfall.

For leaf and ear damage, we first re-scaled the scores from 0 to 8 so that 0 = no damage. Then we used a Poisson model with a log-link function. We used the following independent variables: season, scouting time, landscape type, and *S. frugiperda* infestation.

For the grain yield, we used a Gaussian distribution with an identity link function and the following independent terms: season, landscape type, early damage, and late damage.

For each model, we started with the maximal model and removed nonsignificant terms, starting with the higher level terms while respecting the principle of marginality. We compared models using AIC and retained the model with the lowest Akaike Information Criterion (AIC). For most terms, we assessed the probability of Type I errors based on a two-tailed distribution, but in the case of landscape type we used a one-tailed test (Hypothesis: Higher tree cover → lower *S. frugiperda* infestation and leaf damage and higher yield).

All analyses were conducted in R 3.6.3 (R Core Team 2020). We used the package *lme4* for modeling and the package *ggplot2* for graphing results. We checked our binomial and Poisson models for over-dispersion, and for all models plotted the square-root of the residuals against the fitted values to check for heteroscedasticity.

## Results

### Infestation Rates

**Fig. 1.**
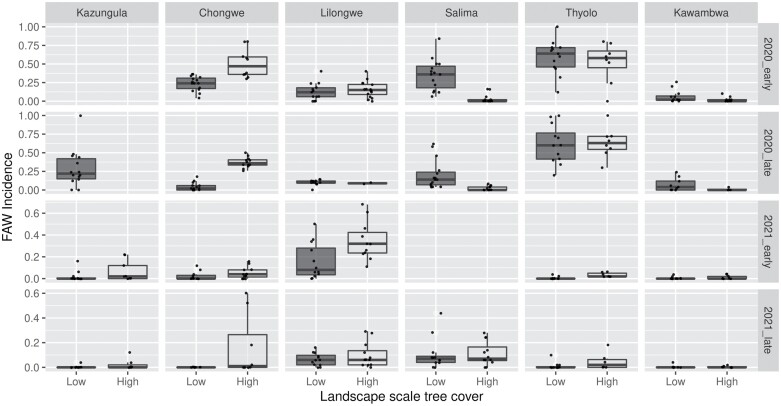
Proportion of infested plants per plot (*S. frugiperda* (FAW) incidence) across landscapes with high and low tree cover. Scouting was conducted at 3–6 wk (early) and 6–9 wk (late) in the 2019–2020 (2020) and 2020–2021 (2021) seasons. Sites are arranged from left to right in order of increasing mean annual rainfall. Missing plots/bars indicate missing data.

*S. frugiperda* infestation rates varied substantially among sites, landscapes, and seasons (Fig. 1, Supp Table 1 [online only]). In the 2019/2020 season, the proportion of plants infested with *S. fruigiperda* in a plot varied from 0.00 to 1.00, while the median plot-level infestation varied from 0.00 to 0.57 across landscapes. The corresponding figures for the 2020/2021 season were 0.00–0.69 and 0.00–0.32. Infestation rates were lower in high tree cover landscapes, in the late season scouting, and in the 2020/2021 season (Fig. 1, Supp Table 1 [online only]). In addition, there were significant interactions between rainfall and year, rainfall and scouting time, landscape tree cover and year, and landscape tree cover and scouting time. Thus, *S. frugiperda* infestation was always low at Kawambwa, which was the highest rainfall site, but the pattern across other sites was inconsistent. The effects of scouting time and landscape tree cover were reduced in the second season, apparently reflecting the overall reduction in infestation rates.

### Leaf Damage

**Fig. 2.**
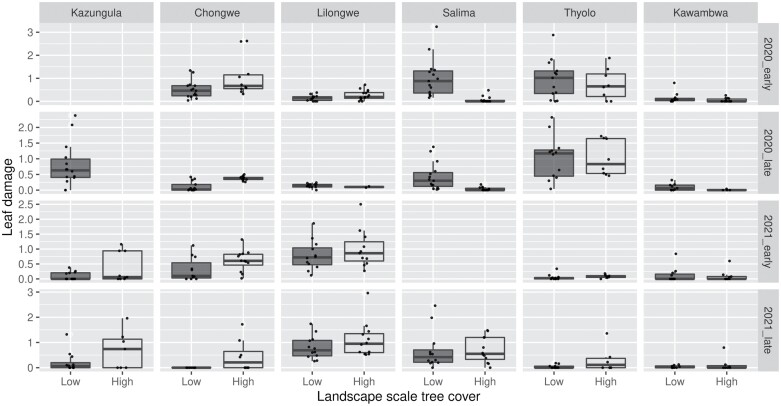
Plot mean leaf damage score per plot across landscapes with high and low tree cover. Damage score was assessed on the Davis 1–9 scale and rescale from 0 to 8, so that 0 = no damage. Scouting was conducted at 3–6 wk (early) and 6–9 wk (late) in the 2019–2020 (2020) and 2020–2021 (2021) seasons. Sites are arranged from left to right in order of increasing mean annual rainfall. Missing plots/bars indicate missing data.

Results for leaf damage were broadly similar to those of infestation (Fig. 2). In the 2019/2020 season, plot level mean leaf damage score (adjusted 0–8 scale) varied from 0.0 to 3.2, while the median of plot-level mean damage varied from 0.0 to 1.4 across landscapes. The corresponding figures for the second season were 0.0–3.0 and 0.0–0.9. Leaf damage scores were lower in high tree cover landscapes, in the late season scouting and in the 2020/2021 season (Supp Table 3 [online only]). There were significant interactive effects between rainfall and year, rainfall and scouting time, landscape tree cover and year, landscape tree cover and scouting time, and year and scouting time. The patterns were similar to those reported for *S. frugiperda* infestation.

Looking at severe leaf damage (scores 5–8), the first noteworthy finding is that the proportion of plots with severely damaged plants was remarkably low (Table 2). For example, 84% of plots did not have any severely damaged plants and only 3% of plots had ≥10% severely damaged plants. Model results for severe leaf damage were similar to leaf damage except that landscape tree cover had a substantial effect in reducing severe leaf damage (Supp Table 3 [online only]).

**Table 2.** Proportion of plots with different percentages of severely damaged plants (adjusted Davis score 4–8) across scouting periods and seasons

	≥2%	≥5%	≥10%	≥20%
Early 2020	0.153	0.061	0.038	0.015
Late 2020	0.086	0.017	0.009	0.009
Early 2021	0.198	0.085	0.028	0.000
Late 2021	0.296	0.113	0.061	0.009

*S. frugiperda* infestation was a significant predictor of leaf damage, but the adjusted *r*^2^ was only 0.51, and only 0.06 for severe leaf damage.

### Yield

**Fig. 3.**
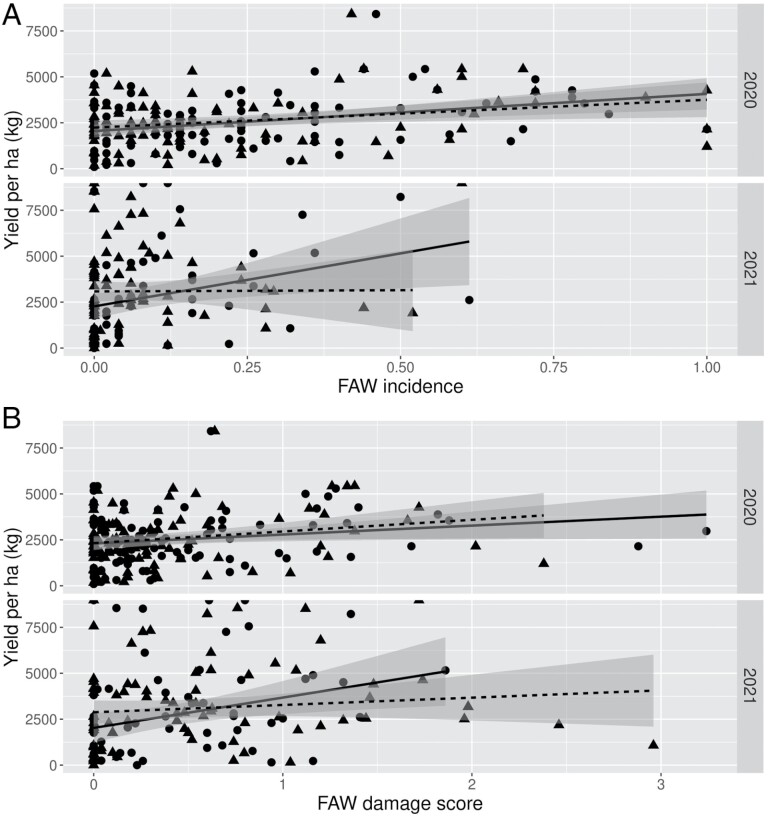
Yield per ha (kg) against (A) *S. frugiperda* incidence and (B) *S. frugiperda* leaf damage score for the 2019–2020 (2020) and 2020–2021 (2021) seasons. Circles and solid trend line indicate data from the early scouting period (3–6 weeks after emergence) and triangles and dashed trend line represent data from the late scouting period (6–9 weeks after emergence). Maize yield could not be predicted from *S. frugiperda* incidence or *S. frugiperda* leaf damage scores.

Despite our standardised experimental approach, the removal of failed plots, and statistically controlling for differences among sites, there was a huge variation in yields among plots. There were no significant relationships between either *S. frugiperda* incidence (%) (Fig. 3A) or leaf damage severity (Fig. 3B) and yield. Relative to the other sources of variance in yield, the impact of *S. frugiperda* was small.

## Discussion

Our dataset is derived from a large-scale trial, covering a wide range of rain-fed maize growing conditions. Our trials were established across six sites in Malawi and Zambia, with two landscapes per site and 12–15 farms per landscape, and repeated over two years. Plots were established using standard protocols and we used appropriate seed varieties and fertiliser applications for the local conditions. In short, this was a well replicated, large-scale trial that is well placed to assess the impact of *S. frugiperda* on smallholders’ fields in Malawi and Zambia.

We found that *S. frugiperda* infestation levels varied from zero to 1.00 at the plot scale, while median of plot infestation rates varied from zero to 0.57 across landscapes. At the high end of the scale, our results fall within the range of values reported from around the region. However, in our trial a large number of plots had very low infestation (Fig. 1). We also found low levels of leaf damage in most plots (Fig. 2). Maize plants have a high capacity to recover from leaf damage (Hruska 2019) and hence leaf damage scores of less than Score 4 (<5 on the original Davis scale) are unlikely to have any impact on yield. A more informative metric may be the proportion of severely damaged plants (Table 2). However, 84% of our plots did not have any severely damaged plants and only 3% of plots had ≥10% severely damaged plants. This suggests that a large proportion of *S. frugiperda* larvae are dying before they reach the fifth and sixth instars, which cause most of the damage (at low levels of infestation larvae may also move among plants). Unsurprisingly, therefore, infestation rate was not a reliable predictor of severe leaf damage (*r*^2^ = 0.06).

After controlling for variance among sites, we found that *S. frugiperda* infestation rates and leaf damage scores were lower in high tree cover landscapes, the late season scouting, and in the second year. In the second year the effect of scouting time was less pronounced, but this can be explained by the fact that the *S. frugiperda* infestation rates and damage scores were low and hence there was limited variance (Figs. 1 and 2). Rainfall, especially heavy rain, is known to be an important mortality factor for *S. frugiperda* (Varella et al. 2015). Young larvae may be washed off leaves and old larvae can drown in the whorl. Wet conditions may also promote higher rates of infection with entopathogenic fungi (Guo et al. 2020). Hence, finding that our wettest site had very low infestation and leaf damage aligns with expectations. In addition, we found that high tree cover landscapes had significantly lower rates of infestation and leaf damage. Landscapes with higher tree cover may be expected to deliver enhanced ecosystem services through harbouring more plant biodiversity (Wan et al. 2020). This may include insectivorous bats (Maas et al. 2016), which are important predators of the adult *S. frugiperda* moths, and arthropod natural enemies (Wan et al. 2020). Clarkson et al. (2022) also recently found that proximity to forest reduced *S. frugiperda* infestation in Zimbabwe. These results underline the importance of conserving areas of (semi-)natural vegetation in agricultural landscapes for pest control services. We recorded a decline in incidence of infestation and leaf damage severity from the early to the late season scouting. Variation in the proportion of larvae versus other stages of the life-cycle (i.e., pupae or adults) can drive temporal variation in infestation and damage, but as our sites support overlapping generations this is unlikely to have a substantial effect. Rather, the declining impact of *S. frugiperda* through the season is most likely caused by natural mortality.

We could not predict maize yield from infestation or leaf damage, even when we used data from both scouting assessments (Fig. 3). Maize has a tremendous capacity to recover from leaf damage (Hruska 2019) and it is often difficult to determine yield loss in carefully controlled screen-house trials, let alone on farmers’ fields. Differences in soils among farmers’ plots and among farmers in their management of the plots contributed to high background variance in yields, even though we standardised the planting protocols and fertiliser application rates. Other pests and diseases may also have contributed, but the rates of infestation with Maize streak virus (6.1%) and stemborers (0.2%) were low. On the one hand, this indicates that for smallholders far bigger yield gains can be achieved through improved soil management and crop husbandry, than through enhanced *S. frugiperda* control. On the other hand, this places us in a quandary when trying to develop action thresholds for IPM. Our results, and those from other studies (Baudron et al. 2019), indicate that infestation rates are not a reliable indicator of yield loss and, contrary to prevailing advice (McGrath et al. 2018), should not be used. In the 2019/2020 season, 40% of our plots exceeded the early season threshold for action (i.e., 20% infestation) and 17.6% exceeded the late season threshold for action (i.e., 40% infestation). In the 2020/2021 season the figures were 10% and 2.2%, respectively. However, very few if any of our plots suffered sufficient impact to justify spraying. A large, but variable, proportion of the *S. frugiperda* population occurring in a field will suffer from natural mortality, so the infestation rate imparts limited useful information. We suggest focusing on the proportion of severely damaged plants, because this serves not only as a measure of damage but also an index of the number of larvae that are surviving to the late stage instars. In addition, we found that infestation level and leaf damage declined from the first to the second scouting. Hence, action thresholds should probably be based on at least two scouting assessments. A useful metric could, e.g., be the trend in the proportion of severely damaged plants from the first to second assessment.

The levels of *S. frugiperda* infestation and leaf damage we recorded suggest a minimal impact on smallholders’ fields in Malawi and Zambia. Against the background variance caused by differences in soils and plot management, we were unable to detect any significant effect of *S. frugiperda* infestation, or leaf damage on yield. These results are in stark contrast to many other reports from the region, including from Malawi and Zambia, that have tended to suggest very high levels of yield loss caused by *S. frugiperda* (Kansiime et al. 2019, De Groote et al. 2020, Kassie et al. 2020, Abro et al. 2021, Tambo et al. 2021, Kwasi et al. 2022, Makale et al. 2022). How can we reconcile these very different findings? Estimates based on farmer perceptions have been found to exaggerate *S. frugiperda* impacts (Baudron et al. 2019). In addition, the metric used in social survey based studies is often simply *S. frugiperda* affected versus non-*S. frugiperda* affected households (De Groote et al. 2020, Kassie et al. 2020, Tambo et al. 2021, Kwasi et al. 2022), which is clearly problematic. If *S. frugiperda* is present in an area, then at the scale of a smallholder property (~1 ha) one would anticipate close to 100% infestation. Thus, reported results may reflect geographically driven variation in yields that have nothing to do with *S. frugiperda*. Alternatively, the farmers may be parsing the metric somewhat to separate heavily affected fields from less affected fields, but with unclear boundaries. However, if a field in a particular area is badly affected by *S. frugiperda* compared to neighbors, this may well be driven by poor management, such as late planting or poor weeding (Baudron et al. 2019). So it is then unclear whether the metric is measuring the impact of *S. frugiperda* or variation in management quality. In short, unless data on *S. frugiperda* infestation, damage, and yield loss are collected through field measurements, we cannot reliably assign differences in household income or food security to *S. frugiperda*.

More generally, although our study covered a wide range of agroecological conditions, it obviously did not encompass all possible situations. For example, we are aware of some ‘hot-spot’ areas in Zambia and Malawi. These appear to be where winter maize is grown using irrigation from permanent streams, such as in Chirundu district along the banks of the Zambezi, thereby maintaining a high population of *S. frugiperda* through the dry season. In other countries, where there are two cropping seasons or a single extended maize growing season, populations of *S. frugiperda* may be able to build up to high levels and escape their natural enemies. It is also possible that immediately following its arrival in sub-Saharan Africa the impacts of *S. frugiperda* were higher and that they have subsequently subsided as natural enemies have learnt to attack the pest. Unfortunately, we do not know because reliable data are lacking. Our results suggest the impacts of *S. frugiperda* may have been overestimated in many places across the continent.

## Supplementary Material

toac113_suppl_Supplementary_Material

## Notes

A paper invited for consideration for the Fall Armyworm special collection.
