# Supplementary material for: Low Impact of Fall Armyworm (Spodoptera frugiperda Smith) (Lepidoptera: Noctuidae) Across Smallholder Fields in Malawi and Zambia
Source: J Econ Entomol. 2022 Dec 14;115(6):1783–9. doi: 10.1093/jee/toac113 (PMC9748589; doi:10.1093/jee/toac113)
Supplement: Supplementary file 1 [file toac113_suppl_supplementary_material.zip › toac113_suppl_Supplementary_Material.odt]

Supplementary Materials

Table S1 Model effects table for Fall armyworm infestation. The model was a binomial GLMM with a log link function. The random term was (1 | Site/Landscape_type/Farmer_name). The dispersion factor was 0.798.

|  | Estimate | SD error | Z value | P value |
| --- | --- | --- | --- | --- |
| Intercept | 1.382 | 1.670 | 0.828 | 0.4077 |
| Rainfall | -0.002 | 0.002 | -1.493 | 0.1354 |
| Landscape tree cover | -0.981 | 0.488 | -2.008 | 0.0223 |
| Scouting time | -2.030 | 0.269 | -7.551 | 0.0000 |
| Year | -2.108 | 0.327 | -6.456 | 0.0000 |
| Rainfall:Year | -0.001 | 0.000 | -3.029 | 0.0025 |
| Rainfall:Scouting | 0.002 | 0.000 | 6.037 | 0.0000 |
| Landscape:Scouting | 0.579 | 0.089 | 6.503 | 0.0000 |
| Landscape:Year | 1.859 | 0.119 | 15.665 | 0.0000 |

Table S2 Model effects table for FAW leaf damage. Leaf damage was measured on a scale of 0-8, where 0 = no damage. We modelled leaf damage using a GLMM with Poisson distribution and a log link function.  The random term was (1 | Site/Landscape_type/Farmer_name). The dispersion factor was 1.090.

|  | Estimate | SD error | Z value | P value |
| --- | --- | --- | --- | --- |
| Intercept | 1.110 | 1.151 | 0.965 | 0.3347 |
| Rainfall | -0.002 | 0.001 | -1.762 | 0.0781 |
| Landscape tree cover | -0.842 | 0.305 | -2.757 | 0.0029 |
| Scouting time | -0.612 | 0.134 | -4.569 | 0.0000 |
| Year | -0.778 | 0.140 | -5.569 | 0.0000 |
| Rainfall:Year | -0.000 | 0.000 | -2.758 | 0.0058 |
| Rainfall:scouting | 0.000 | 0.000 | 2.219 | 0.0026 |
| Landscape:Year | 1.312 | 0.050 | 26.096 | 0.0000 |
| Landscape:Scouting | 0.210 | 0.044 | 4.784 | 0.0000 |
| Scouting:Year | 0.441 | 0.046 | 9.603 | 0.0000 |

Table S3 Model effects table for FAW severe leaf damage (Davis Score 4-8). Severe leaf damage was modelled using a GLMM with a binomial response (not severely damaged, severely damaged).  The random term was (1 | Site/Landscape_type/Farmer_name). The dispersion factor was 0.314.

|  | Estimate | SD error | Z value | P value |
| --- | --- | --- | --- | --- |
| Intercept | -2.647 | 1.221 | -2.168 | 0.0301 |
| Rainfall | -0.002 | 0.001 | -1.621 | 0.1049 |
| Landscape tree cover | -1.536 | 0.392 | -3.921 | 0.0000 |
| Scouting time | -1.322 | 0.255 | -5.191 | 0.0000 |
| Year | -3.188 | 0.896 | -3.558 | 0.0004 |
| Rainfall:Year | 0.002 | 0.001 | 2.609 | 0.0091 |
| Landscape:Year | 2.705 | 0.361 | 7.501 | 0.0000 |
| Scouting:Year | 1.432 | 0.311 | 4.600 | 0.0000 |
